# Australian Aboriginal children have higher hospitalization rates for otitis media but lower surgical procedures than non-Aboriginal children: A record linkage population-based cohort study

**DOI:** 10.1371/journal.pone.0215483

**Published:** 2019-04-23

**Authors:** Darren W. Westphal, Deborah Lehmann, Stephanie A. Williams, Peter C. Richmond, Francis J. Lannigan, Parveen Fathima, Christopher C. Blyth, Hannah C. Moore

**Affiliations:** 1 Wesfarmers Centre for Vaccines and Infectious Diseases, Telethon Kids Institute, The University of Western Australia, Perth, Western Australia, Australia; 2 National Centre for Epidemiology & Population Health, Australian National University, Canberra, Australian Capital Territory, Australia; 3 Division of Paediatrics, School of Medicine, The University of Western Australia, Perth, Western Australia, Australia; 4 Division of Surgery, Paediatrics and Child Health, The University of Western Australia, Perth, Western Australia, Australia; 5 Sidra Medicine, Doha, Qatar; 6 Department of Infectious Diseases, Perth Children’s Hospital, Perth, Western Australia, Australia; 7 Department of Microbiology, PathWest Laboratory Medicine, QEII Medical Centre, Perth, Western Australia, Australia; National Center for Global Health and Medicine, JAPAN

## Abstract

**Introduction:**

Otitis media (OM) is one of the most common infectious diseases affecting children globally and the most common reason for antibiotic prescription and paediatric surgery. Australian Aboriginal children have higher rates of OM than non-Aboriginal children; however, there are no data comparing OM hospitalization rates between them at the population level. We report temporal trends for OM hospitalizations and in-hospital tympanostomy tube insertion (TTI) in a cohort of 469,589 Western Australian children born between 1996 and 2012.

**Materials and methods:**

We used the International Classification of Diseases codes version 10 to identify hospitalizations for OM or TTI recorded as a surgical procedure. Using age-specific population denominators, we calculated hospitalization rates per 1,000 child-years by age, year and level of socio-economic deprivation.

**Results:**

There were 534,674 hospitalizations among 221,588 children hospitalized at least once before age 15 years. Aboriginal children had higher hospitalization rates for OM than non-Aboriginal children (23.3/1,000 [95% Confidence Interval (CI) 22.8,24.0] vs 2.4/1,000 [95% CI 2.3,2.4] child-years) with no change in disparity over time. Conversely non-Aboriginal children had higher rates of TTI than Aboriginal children (13.5 [95% CI 13.2,13.8] vs 10.1 [95% CI 8.9,11.4]). Children from lower socio-economic backgrounds had higher OM hospitalization rates than those from higher socio-economic backgrounds, although for Aboriginal children hospitalization rates were not statistically different across all levels of socio-economic disadvantage. Hospitalizations for TTI among non-Aboriginal children were more common among those from higher socio-economic backgrounds. This was also true for Aboriginal children; however, the difference was not statistically significant. There was a decline in OM hospitalization rates between 1998 and 2005 and remained stable thereafter.

**Conclusion:**

Aboriginal children and children from lower socio-economic backgrounds were over-represented with OM-related hospitalizations but had fewer TTIs. Despite a decrease in OM and TTI hospitalization rates during the first half of the study for all groups, the disparity between Aboriginal and non-Aboriginal children and between those of differing socioeconomic deprivation remained.

## Introduction

Otitis media (OM) is one of the most common infectious diseases affecting children [1]. Approximately two-thirds of Australian children will have at least one episode of OM by the time they reach their first birthday [2]. Incidence is most common among children aged 18–24 months [3]. OM is the most common reason for antibiotic prescription,[4] thus contributing to increased antibiotic resistance in the common bacterial pathogens responsible for OM [5]. OM also leads to the most commonly performed surgery in children, namely tympanostomy tube insertion (TTI), as well as adenoidectomy or adenotonsillectomy [6]. TTI is recommended for management of recurrent acute OM (AOM) or persistent bilateral OM with effusion (OME) with hearing loss.

Globally, children in resource-poor settings have the highest documented rates of AOM [7]. Australian Aboriginal and/or Torres Strait Islander children (hereafter referred to as Aboriginal) have some of the highest rates of OM in the world [8]. In a birth cohort study conducted between 1999 and 2005 in the remote Goldfields region of Western Australia (WA), OM was detected in 55% of examinations of Aboriginal children and 26% in examinations of non-Aboriginal children aged less than 2 years [9].

The seven-valent pneumococcal conjugate vaccine (7vPCV) was introduced and funded as a 3+0 infant dose schedule in Australia in 2001 for Aboriginal children (with a pneumococcal polysaccharide vaccine booster at 18 months of age). In 2005, 7vPCV was introduced for all Australian children through the National Immunisation Program at 2, 4 and 6 months of age [10]. In 2011, 7vPCV was replaced with the 13-valent PCV (13vPCV) and for high risk children, a fourth dose of 13vPCV replaced the existing pneumococcal polysaccharide vaccine. Following the introduction of PCV in some countries, including Australia, there was evidence demonstrating protection against OM due to *Streptococcus pneumoniae*, one of the three otopathogens responsible for OM [11]. However, more recently, that protection may have been compromised by serotype replacement with an increase in non-vaccine serotypes observed [12].

The majority of cases of OM are managed in the community by general practitioners. Nevertheless, there is limited information on the severe clinical burden of OM in terms of hospitalizations in the general Australian population [2] and no information comparing OM-related hospitalizations between non-Aboriginal and Aboriginal children at the population level. We used linked administrative data to investigate the overall morbidity and trends in hospitalizations for OM and OM-related procedures in a total population birth cohort of children over a 16-year period. Our primary aim was to describe the age-specific temporal trends of hospitalization for OM and TTI in non-Aboriginal and Aboriginal children. Our secondary aims were to describe the hospitalization rates for OM and TTI by region of birth and level of socio-economic disadvantage.

## Materials and methods

### Setting and population

WA covers the western third of Australia and is sparsely populated with 2.5 million residents, 1.9 million of whom live in the metropolitan capital, Perth. WA is divided into seven administrative regions for provision of medical and public health services. These regions were grouped into metropolitan, rural, and remote based on Western Australia Department of Health classifications that take into account the population density of the region and distance to the nearest services [13].

Aboriginal people account for approximately 4% of the WA population [14]; however, higher proportions of Aboriginal people reside in rural and remote regions in comparison to non-Aboriginal people. In 2012, children and young people aged 0–17 years comprised 22.9% of the state's population, 73% of whom were living in metropolitan areas, 17% in rural areas and 10% in remote regions [15].

### Data source and coding

We analyzed individual-level linked population data that were extracted and probabilistically linked through the WA Data Linkage System [16]. We identified a birth cohort of 469,589 live born children between 1996 and 2012 through the Midwives’ Notification System (containing information on approximately 99% of births occurring in WA) and the Birth and Death registers. All hospital admission records for the cohort were extracted from the Hospital Morbidity Data Collection, containing information on all inpatient separations and day admissions from all public and private hospitals in the state.

Hospital records with an admission and separation date between 1996 and 2012 were identified (herein referred to as hospitalizations). Hospitalization data were available through to 31 December 2012; thus, our dataset contained information on hospitalizations for children aged up to 15 years. We used the International Classification of Diseases, Ninth/Tenth Revision, Clinical/Australian Modification (ICD-9-CM or ICD-10-AM) [17] to identify hospital admissions with relevant OM-related codes listed in the principal or in any of the 20 additional diagnosis fields and included H65-67, H70-75, H90.0-H90.2 and H90.6-H90.8, H92, and/or H95. The principal diagnosis was related to the condition that required the most care during the hospitalization. Additional diagnoses were those related to other conditions that also required care. For the purpose of this analysis, an OM episode was defined as having a relevant principal and/or additional diagnosis code where an OM procedure was not performed during the same hospitalization. We chose to combine principal and additional diagnoses to better capture the full burden of OM-related hospitalizations. TTI was defined by a principal or additional procedure coded as 41626–01 or 41632–01. In Australia, all TTIs are performed in hospitals on inpatients, and thus have a recorded admission.

Inter- and intra-hospital transfers were merged into a single admission. Hospitalizations for OM occurring within 14 days of a previous OM admission were considered part of the same illness episode and were combined.

### Statistical methods

We calculated overall and annual age-specific hospitalization rates per 1,000 child-years for OM diagnoses and TTI, using person-time-at-risk as the denominator. For TTI, we applied a three-year moving average to smooth out annual variations. All analyses were conducted separately for Aboriginal and non-Aboriginal children and by region of birth using residential postal codes collected at birth. Incidence rate ratios (IRR) and 95% confidence intervals (CI) were used to compare the rates for Aboriginal and non-Aboriginal children. We used the following age groups in the analysis: <6, 6–11, 12–17, 18–23 months and 2, 3–4, 5–9, 10–14 years.

We used a score from the index of relative socio-economic advantage and disadvantage (IRSAD), one of the four indices from the Socio-Economic Index For Australia (SEIFA), derived from the Australian Bureau of Statistics [18]. The IRSAD score is derived from 21 different variables which include low or high income, internet connection, skilled or unskilled occupations and education [18]. The IRSAD scores used in our analyses were based on the mother’s residential address at the time of her child’s birth and scored at the collector’s district level, an area of approximately 200 dwellings and the smallest unit available for population-based analyses. The scores were grouped into five categories ranging from most disadvantaged (<10% of the index scores) to most advantaged (>90% of the index scores). For analyses of OM disease and TTI according to socio-economic categories, only hospitalizations for OM before age 2 years and TTI before age 5 years are included, as these were the age groups with the highest incidence. Any cell counts with fewer than five observations are reported as <5. All analyses were completed using Stata 14.1 (Stata Corp. College Station, TX).

### Ethics statement

Approval for this study was granted by the Department of Health Western Australia Human Research Ethics Committee, the Western Australian Aboriginal Health Ethics Committee, the University of Western Australia Human Research Ethics Committee and the Australian National University Human Research Ethics Committee.

## Results

**Fig 1:**
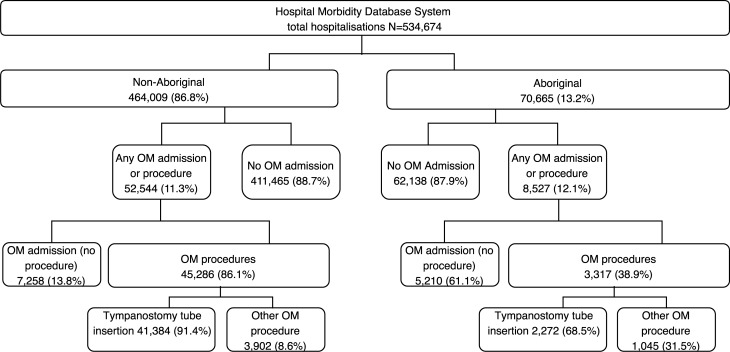
Flow chart of record linkage of a cohort of 469,589 children born in Western Australia between 1996 and 2012 with records of hospitalization for otitis media or tympanostomy tube insertion procedure before 15 years of age.

There were 31,348 (6.7%) Aboriginal children and 240,237 (51.2%) boys from the overall cohort of 469,859 births between 1996 and 2012. Singleton births accounted for 455,675 (97.0%) of the cohort and 2,538 (0.5%) children had died by the end of the study period. There were 534,674 hospitalization episodes recorded for 221,588 children hospitalized at least once before the age of 15 years. There were 70,665 (13.2%) hospitalizations for Aboriginal children. OM (excluding procedures) accounted for 7,258 (1.6%) of all hospitalizations among non-Aboriginal and 5,210 (7.4%) among Aboriginal children (Fig 1). Among these, OM hospitalizations were coded as the principal diagnosis in 2,437/7,258 (33.6%) of non-Aboriginal and 1,292/5,210 (24.8%) of Aboriginal children. In S1 Table we list the primary diagnoses when only an additional diagnosis field had an OM code.

There were 48,603 OM-related procedures, 43,656 (89.8%) of which were for TTI. TTIs accounted for 41,384/464,009 (8.9%) of admissions among non-Aboriginal and 2,272/70,665 (3.2%) of admissions among Aboriginal children. These included repeat admissions for TTI; for non-Aboriginal children, 31.1% had more than one hospitalization for TTI (range 2–10), while 24.5% of Aboriginal children had repeat hospitalizations for TTI (range 2–8).

### Hospitalization age-specific rates and temporal trends

**Fig 2:**
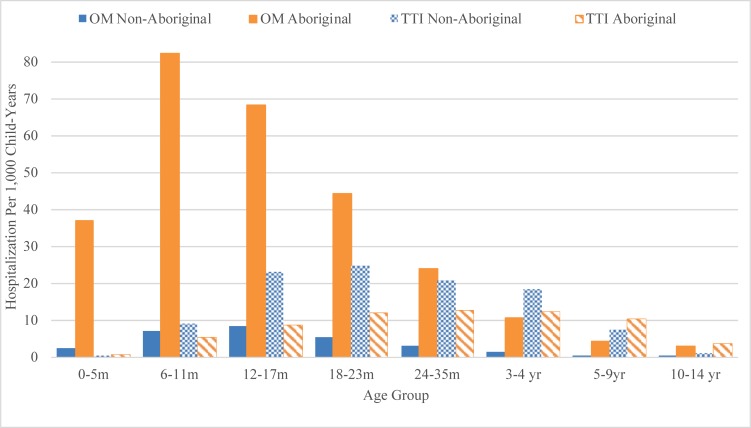
Rates of hospitalization for non-procedural otitis media and tympanostomy tube insertion per 1,000 child-years for non-Aboriginal and Aboriginal children born in Western Australia between 1996 and 2012.

The overall OM admission rate from 1996 to 2012 in children aged <15 years was 2.4/1,000 child-years (95% CI: 2.3,2.4) among non-Aboriginal and 23.3/1,000 child-years (95% CI: 22.8, 24.0) among Aboriginal children (Table 1). Compared to other age groups, Aboriginal children aged 6–11 months had the highest non-procedural OM hospitalization rates while non-Aboriginal children aged 12–17 months had the highest rates. Aboriginal children experienced higher hospitalization rates than non-Aboriginal children across all age groups, with the greatest disparity seen in the 0–5-month age group (Fig 2 and Table 1). A total of 1,517/7,258 (20.9%) non-Aboriginal and 2,034/5,210 (39.1%) Aboriginal children had >1 OM-related hospitalization. The median age of first hospitalization was 18.3 months among non-Aboriginal children (range: 1.4 months to 14 years) compared with 15.3 months (range: 8 days to 13 years) among Aboriginal children.

**Table 1:** Hospitalization rates for non-procedural otitis media (per 1,000 child-years) with 95% confidence intervals in a Western Australian birth cohort 1996–2012, by Aboriginal status and region of birth*.

	Non-Aboriginal			Aboriginal			IRR (95% CI)
Age	No.	Rate^†^	Regional IRR (95% CI)	No	Rate^†^	Regional IRR (95% CI)	Aboriginal: non-Aboriginal
**0–5 months**							
Metropolitan	281	1.7	Reference	80	14.6	Reference	**8.6 (6.6,11.1)**
Rural	124	3.6	**2.1 (1.7,2.6)**	98	27.1	**1.9 (1.4,2.5)**	**7.6 (5.8,10.0)**
Remote	62	4.5	**2.7 (2.0,3.5)**	414	67.7	**4.6 (3.6,6.0)**	**15.1 (11.5,20.0)**
**6–11 months**							
Metropolitan	880	5.5	Reference	191	36.3	Reference	**6.6 (5.6,7.7)**
Rural	368	11.0	**2.0 (1.8,2.2)**	210	60.3	**1.7 (1.4,2.0)**	**5.5 (4.6,6.5)**
Remote	167	12.5	**2.3 (1.9,2.7)**	887	150.0	**4.1 (3.5,4.9)**	**12.0 (10.2,14.2)**
**12–17 months**							
Metropolitan	1,048	6.9	Reference	154	30.4	Reference	**4.4 (3.7,5.3)**
Rural	355	10.9	**1.6 (1.4,1.8)**	181	53.7	**1.8 (1.4,2.2)**	**4.9 (4.1,5.9)**
Remote	196	15.2	**2.2 (1.9,2.6)**	692	121.0	**4.0 (3.3,4.8)**	**8.0 (6.8,9.4)**
**18–23 months**							
Metropolitan	686	4.7	Reference	96	19.6	Reference	**4.2 (3.4,5.2)**
Rural	218	6.9	**1.5 (1.3,1.7)**	109	33.5	**1.7 (1.3,2.3)**	**4.8 (3.8,6.1)**
Remote	109	8.7	**1.9 (1.5,2.3)**	424	76.6	**3.9 (3.1,4.9)**	**8.6 (7.0,10.7)**
**24–35 months**							
Metropolitan	711	2.6	Reference	90	9.7	Reference	**3.8 (3.0,4.7)**
Rural	263	4.4	**1.7 (1.5,2.0)**	131	21.3	**2.2 (1.7,2.9)**	**4.8 (3.9,6.0)**
Remote	94	4.0	**1.5 (1.2,1.9)**	426	40.6	**4.2 (3.3,5.3)**	**10.3 (8.2,13.0)**
**3–4 years**							
Metropolitan	631	1.3	Reference	74	4.5	Reference	**3.5 (2.7,4.4)**
Rural	217	2.0	**1.6 (1.3,1.8)**	121	11.1	**2.5 (1.8,3.3)**	**5.4 (4.3,6.8)**
Remote	74	1.7	**1.3 (1.0,4.7)**	328	17.5	**3.9 (3.0,5.1)**	**10.1 (7.8,13.1)**
**5–9 years**							
Metropolitan	448	0.5	Reference	70	2.5	Reference	**4.6 (3.6,6.0)**
Rural	154	0.8	**1.5 (1.3,1.8)**	74	3.9	**1.6 (1.1,2.2)**	**4.9 (3.6,6.5)**
Remote	44	0.6	1.1 (0.8,1.5)	245	7.2	**2.9 (2.2,3.9)**	**12.9 (9.3,18.1)**
**10–14 years**							
Metropolitan	92	0.2	Reference	18	1.4	Reference	**6.0 (3.4,10.0)**
Rural	19	0.2	0.9 (0.5,1.4)	16	1.9	1.3 (0.6,2.8)	**9.2 (4.4,19.0)**
Remote	<5	0.1	0.4 (0.1,1.2)	56	3.6	**2.6 (1.5,4.6)**	**35.3 (13.0,134.0)**
**Total**							
Metropolitan	4,777	1.8	Reference	773	8.8	Reference	**4.9 (4.5,5.2)**
Rural	1,718	2.9	**1.6 (1.5,1.7)**	940	16.1	**1.8 (1.7,2.0)**	**5.5 (5.1,5.9)**
Remote	750	3.2	**1.7 (1.6,1.9)**	3,472	34.0	**3.9 (3.6,4.2)**	**10.7 (10.4,11.1)**
**Overall**	7,245	2.4	**—**	5,185	23.3	**—**	**9.8 (9.5,10.2)**

*Abbreviations*: Regional IRR, incidence rate ratio of outcome based on region; IRR, incidence rate ratio comparing Aboriginal to non-Aboriginal; 95% CI, 95% confidence interval.

*38 records with missing region of birth were excluded; 13 non-Aboriginal and 25 Aboriginal children.

^†^Rate per 1,000 child-years

Bold indicates statistically significant result at α<0.05

**Fig 3:**
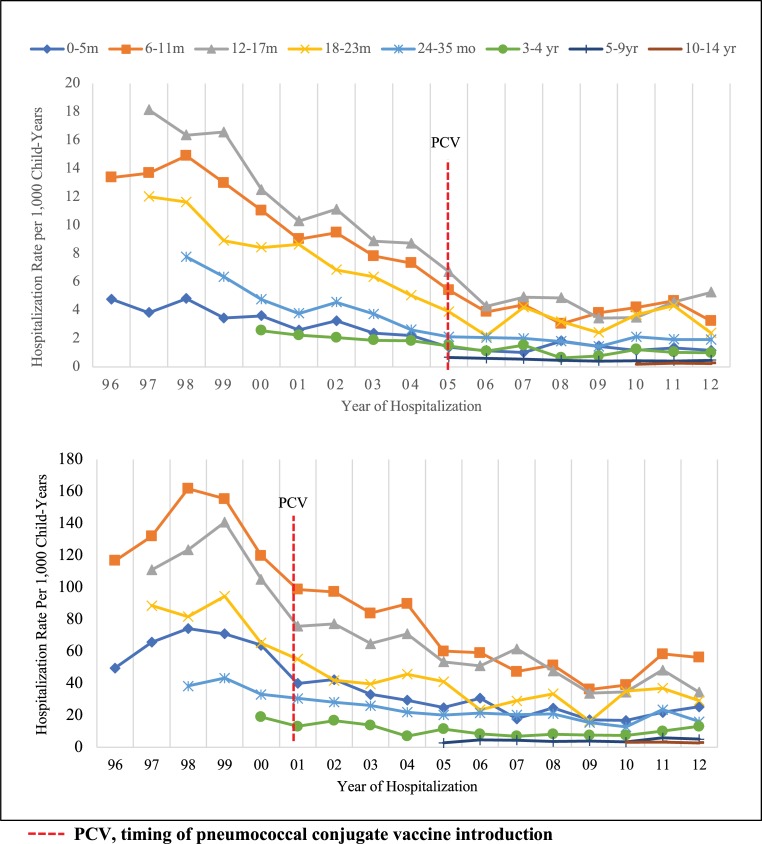
Rates of hospitalization for non-procedural otitis media per 1,000 child-years for non-Aboriginal (top) and Aboriginal (bottom) children born in Western Australia between 1996 and 2012. Note difference in scale.

There was a decline in the OM hospitalization rate from 1998 for both Aboriginal and non-Aboriginal children across all age groups (Fig 3) which stabilized after 2006. There was no obvious impact on OM hospitalization rates after PCV introduction in either population. Aboriginal children aged 6–11 months showed the greatest decline from 161.8/1,000 child-years in 1998 to 56.2/1,000 child-years in 2012. Despite a decline in overall OM hospitalization rates over time in both groups of children, the disparity in rates between the two populations has remained with rates in Aboriginal children approximately 10 times higher than in non-Aboriginal children (Fig 3). Analyses including only those hospitalizations with a principal diagnosis of OM showed similar trends (S1 Fig).

The overall hospitalization rate for TTI was 13.5/1,000 child-years (95% CI 13.4, 13.7) among non-Aboriginal children and 10.1/1,000 child-years (95% CI 9.8, 10.6) among Aboriginal children (Table 2). In 1998, this was highest for non-Aboriginal children aged 12–17 months, (26.7 per 1,000 child-years), 2.8-fold higher than in Aboriginal children of the same age during the same year. By 2011, the highest TTI hospitalization rate for non-Aboriginal children was in children aged 18–23 months at 26.6/1,000 child-years, 2.3-fold higher than among Aboriginal children of the same age. The median age of first TTI admission was 37.2 months for non-Aboriginal (range 43 days to 14.9 years) and 54.9 months (range 8 days to 14.6 years) for Aboriginal children.

**Table 2:** Hospitalization rates for tympanostomy tube insertion (per 1,000 child-years) with 95% confidence intervals in a Western Australian birth cohort 1996–2012, by Aboriginal status and region of birth*.

	Non-Aboriginal			Aboriginal			IRR (95% CI)
Age	No.	Rate^†^	Regional IRR	No.	Rate^†^	Regional IRR	Aboriginal: Non-Aboriginal
**0–5 months**							
Metropolitan	96	0.6	Reference	7	1.3	Reference	2.2 (0.9,4.7)
Rural	10	0.3	**0.5 (0.2,1.0)**	<5	1.1	0.4 (0.0,2.3)	1.9 (0.2,9.0)
Remote	<5	0.3	0.5 (0.1,1.3)	<5	0.3	0.3 (0.03,1.4)	1.1 (0.1,7.9)
**6–11 months**							
Metropolitan	1,620	10.2	Reference	34	6.5	Reference	**0.6 (0.4,0.9)**
Rural	204	6.1	**0.6 (0.5,0.7)**	19	5.5	0.8 (0.5,1.5)	0.9 (0.5,1.4)
Remote	78	5.8	**0.6 (0.5,0.7)**	25	4.2	0.7 (0.4,1.1)	0.7 (0.4,1.2)
**12–17 months**							
Metropolitan	3,957	25.9	Reference	51	10.1	Reference	**0.4 (0.3,0.5)**
Rural	473	14.6	**0.6 (0.5,0.6)**	37	11.0	1.1 (0.7,1.7)	0.8 (0.5,1.1)
Remote	164	12.7	**0.5 (0.4,0.6)**	33	5.8	**0.6 (0.4,0.9)**	**0.5 (0.3,0.7)**
**18–23 months**							
Metropolitan	4,032	27.4	Reference	79	16.2	Reference	**0.6 (0.5,0.8**)
Rural	528	16.8	**0.6 (0.6,0.7)**	42	12.9	0.8 (0.5,1.2)	0.8 (0.6,1.1)
Remote	200	16.3	**0.6 (0.5,0.7)**	42	7.6	**0.5 (0.3,0.7)**	**0.5 (0.3,0.7)**
**24–35 months**							
Metropolitan	6,213	22.5	Reference	153	16.5	Reference	**0.7 (0.6,0.9)**
Rural	976	16.3	**0.7 (0.7,0.8)**	85	13.8	0.8 (0.6,1.1)	0.8 (0.7,1.1)
Remote	301	12.7	**0.6 (0.5,0.6)**	88	8.4	**0.5 (0.4,0.7**)	**0.7 (0.5,0.8)**
**3–4 years**							
Metropolitan	9,589	19.8	Reference	251	15.3	Reference	**0.8 (0.7,0.9)**
Rural	1,648	15.5	**0.8 (0.7,0.8)**	142	13.0	0.9 (0.7,1.1)	0.8 (0.7,1.0)
Remote	555	13.1	**0.7 (0.6,0.7)**	171	9.1	**0.6 (0.5,0.7)**	**0.7 (0.6,0.8)**
**5–9 years**							
Metropolitan	7,889	9.4	Reference	294	10.3	Reference	**1.1 (1.0,1.2)**
Rural	1,708	8.9	1.0 (0.9,1.0)	220	11.6	1.1 (0.9,1.3)	**1.3 (1.1,1.5)**
Remote	560	7.2	**0.8 (0.7,0.8)**	348	10.3	1.0 (0.9,1.2)	**1.4 (1.3,1.6)**
**10–14 years**							
Metropolitan	462	1.2	Reference	45	3.5	Reference	**3.0 (2.1,4.1)**
Rural	108	1.2	1.0 (0.8,1.2)	27	3.1	0.9 (0.5,1.5)	**2.7 (1.7,4.2)**
Remote	37	0.9	0.8 (0.6,1.1)	61	3.9	1.1 (0.8,1.7)	**4.2 (2.7,6.4)**
**Total**							
Metropolitan	33,867	12.9	Reference	914	10.4	Reference	**0.8 (0.8,0.9)**
Rural	5,658	9.7	0.8 (0.7,0.8)	574	9.8	**0.9 (0.9,1.1)**	1.0 (0.9,1.1)
Remote	1,899	8.0	0.6 (0.6,0.7)	772	7.6	**0.7 (0.7,0.8)**	0.9 (0.9,1.0)
**Overall**	41,424	13.5	**—**	2,260	10.1	**—**	**0.7 (0.7–0.8)**

*Abbreviations*: Regional IRR, incidence rate ratio of outcome based on region; IRR, incidence rate ratio comparing Aboriginal to non-Aboriginal children; 95% CI, 95% confidence interval.

^†^Rate per 1,000 child-years

*52 records with missing region of birth were excluded; 40 non-Aboriginal and 12 Aboriginal children.

Bold indicates statistically significant result at α<0.05

**Fig 4:**
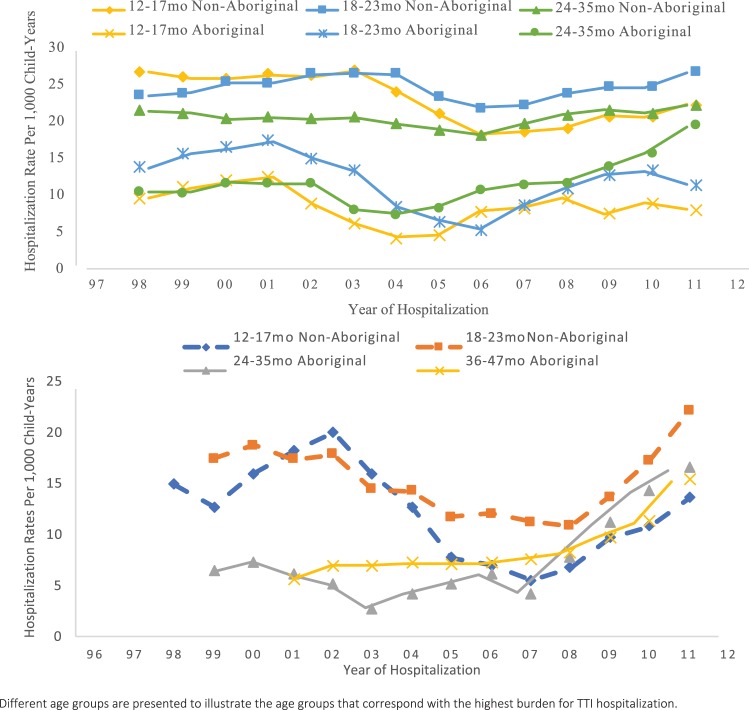
Hospitalization rates for tympanostomy tube insertion among children 12 to 35 months born in Western Australia between 1996 and 2012 (top). Non-Aboriginal children aged between 12 and 23 months and Aboriginal children aged 24 to 47 months born in remote regions in Western Australia between 1996 and 2012. (3-year moving average).

TTI-related hospitalization rates were lower in Aboriginal than non-Aboriginal children between 6 months and 5 years of age but higher in Aboriginal children aged 5–14 years (Table 2). TTI-related hospitalizations fluctuated over time but were generally lowest in the middle of the study period (Fig 4). There was an increase in TTI rates among Aboriginal children aged 24–35 months from 7.3 to 19.4/1,000 child-years between 2003 and 2011.

### Region-specific hospitalization rates

Aboriginal children of all ages from rural or remote areas had rates of non-procedural OM hospitalization 1.9–4.6 times higher than Aboriginal children born in metropolitan areas. For non-Aboriginal children, there was less of a geographic disparity with children aged less than 10 years having hospitalization rates for OM up to 2.7 times higher in children born in rural and remote areas than in children born in the metropolitan area. Hospitalization rates of OM were 4–35 times higher in Aboriginal children than non-Aboriginal children of any age regardless of where they were born, with the greatest disparity seen among 10-14-year-old Aboriginal children born in remote areas (Table 2).

Aboriginal and non-Aboriginal children born in metropolitan Perth had higher TTI rates than those born in rural and remote regions with children aged less than 5 years generally having TTI rates 40–50% lower in rural and remote regions compared to children born in the metropolitan region (Table 2). Overall, 60.2% of all TTIs were performed in metropolitan private hospitals, comprising 63% of all TTIs for non-Aboriginal compared with 7% for Aboriginal children (S2 Table). The increase over time in TTI hospitalization rates noted above was particularly marked for both Aboriginal and non-Aboriginal children born in remote regions. In remote areas of WA, the TTI rate was four times higher among 10–14-year-old Aboriginal children than non-Aboriginal children (IRR 4.2, 95% CI 2.7, 6.4) (Table 2).

### Hospitalization rates by socio-economic deprivation

Non-Aboriginal children in the most socio-economically deprived group had the highest rates of OM-related hospitalizations (Table 3). Although the rates for OM hospitalizations in Aboriginal children were higher in those who were most disadvantaged, they were not statistically different from those in the least disadvantaged areas. All children belonging to the highest socio-economic group had the highest TTI rates, despite having the lowest overall OM-related hospitalizations, however this result was only statistically significant for non-Aboriginal children. OM hospitalization rates were higher and TTI rates lower in Aboriginal than in non-Aboriginal children in each of the socio-economic levels (Table 3).

**Table 3:** Otitis media hospitalization rates among children aged <2 years and TTI among children <5 years at the time of admission according to socio-economic categories and Aboriginal status.

Socio-Economic Group OM	Non-Aboriginal			Aboriginal			IRR Aboriginal:Non-Aboriginal
	Total	Rate^†^	IRR	Total	Rate^†^	IRR	
0–10% (most disadvantaged)	578	9.4	**3.7 (3.1,4.5)**	1,068	64.4	1.4 (0.8,2.7)	**6.9 (6.2,7.6)**
11–25%	886	7.5	**3.0 (2.5,3.6)**	485	39.1	0.9 (0.5,1.6)	**5.2 (4.6,5.8)**
26–75%	1,995	5.2	**2.1 (1.7,2.5)**	790	44.1	1.0 (0.6,1.9)	**8.6 (7.9,9.3)**
76–90%	481	3.9	**1.5 (1.3,1.9)**	57	33.4	0.7 (0.4,1.5)	**8.6 (6.5,11.4)**
91–100% (least disadvantaged)	150	2.5	Reference	13	45.5	Reference	**18.2 (9.5,32.1)**
**TTI**							
0–10% (most disadvantaged)	1,959	14.0	**0.7 (0.6,0.7)**	339	9.1	0.6 (0.3,1.4)	**0.7(0.6,0.7)**
11–25%	4,005	15.3	**0.7 (0.7,0.8)**	270	9.9	0.7 (0.4,1.5)	**0.7 (0.6,0.7)**
26–75%	14,486	16.9	**0.7 (0.7,0.8)**	429	10.6	0.7 (0.4,1.6)	**0.6 (0.6,0.7)**
76–90%	5,351	19.7	**0.9 (0.8,0.9)**	49	13.5	0.9 (0.4,2.1)	**0.7 (0.5,0.9)**
91–100% (least disadvantaged)	2,863	21.4	Reference	9	14.6	Reference	0.7 (0.3,1.3)

*Abbreviations*: OM, otitis media; TTI, tympanostomy tube insertion; IRR, incidence rate ratio

^†^Rate per 1,000 child-years

Bold indicates statistically significant result at α<0.05

## Discussion

To our knowledge, this is the first report of hospitalization rates for OM in a total birth cohort that has examined differences between Aboriginal and non-Aboriginal children and between levels of socio-economic disadvantage. Throughout the study period Aboriginal children were 10 times more likely to be hospitalized for OM than non-Aboriginal children but less likely to have TTI. The disparity remained even when only principal diagnoses were compared (S1 Fig). Furthermore, while hospitalization rates were consistently higher in Aboriginal than non-Aboriginal children, non-Aboriginal children from low socio-economic backgrounds and all children living in rural or remote areas were at increased risk of hospitalization for OM but less likely to have TTI.

Our data show that the median age of OM hospitalization was three months younger among Aboriginal than non-Aboriginal children, consistent with data from field studies [9, 19]. This is notable, since early onset of OM is associated with increased risk of severe OM and complications [1, 20]. Conversely, the median age for first TTI admission was 18 months later for Aboriginal children.

We demonstrated that the burden of OM-related hospitalizations was highest among children born in remote areas of WA, consistent with previous reports in Australia, the US, and Canada [21–23]. Our finding that children in the most disadvantaged socio-economic group experienced the highest rates of OM-related hospitalizations are also consistent with studies in the US [24].

OM hospitalization rates declined between 1998 and 2006 among all WA children, but remained stable thereafter. This could be because the disease became less severe over time due to improvements in nutrition and living conditions or because more children were being treated in the community in later years, prior to requiring hospitalization. The introduction of PCV for Aboriginal children in 2001 may have contributed to the ongoing decline in hospitalization rates in the Aboriginal population (though coverage was low at 45–60%) but does not explain the contemporaneous decline in non-Aboriginal children as herd immunity would have been limited prior to the introduction of a national universal PCV program in 2005. When the universal program began, on-time receipt of 7vPCV was 78% for non-Aboriginal children [25]. Pneumococcal serotype or pathogen replacement with non-typeable *Haemophilus influenzae* following universal introduction of 7vPCV [26] may in part explain the lack of further reduction in OM hospitalization after 2005.

In our study, TTI was conducted at a younger age, generally in private metropolitan hospitals, and more often in non-Aboriginal than in Aboriginal children up to five years of age in all regions of WA. This suggests that factors other than geographic location influence TTI rates, in particular level of socioeconomic deprivation (S2 Table). In an analysis of data from the National Maternal and Infant Health Survey in the USA, Kogan and colleagues [27] found that children who did not have health insurance were less likely to have a TTI than those who did.

It is noteworthy, in our study, that TTI rates in Aboriginal children aged 5–15 years were higher than in non-Aboriginal children. This is likely due to delayed detection of OM until Aboriginal children start school and possibly complicated by shortages of audiology services which would delay assessment [28].

We found TTI rates were lowest between 2004 and 2006. On a positive note, there was an increase between 2006 and 2012, particularly in remote parts of the state. This is in part due to increased ENT services to rural and remote locations in recent years [29] but may also reflects a change in the epidemiology of ear disease, *i*.*e*. now more closed disease (long-standing OME for which TTI is recommended) rather than the more serious chronic suppurative OM (CSOM) characterized by perforation of the tympanic membrane requiring tympanoplasty. Where there is intensive management of middle ear disease there is a corresponding decrease of CSOM and increase in OME [26, 30].

In 2006, Gunasekera *et al* explored the availability of ear health services in Aboriginal Medical Services (AMS) across Australia. They noted practitioners in rural or remote AMSs reported managing a higher load of OM cases and reported fewer specialist health services (*e*.*g*. audiology, ENT surgery, and hearing aids) than practitioners in metropolitan AMSs [31], thus corroborating our results.

In our study, the most socio-economically advantaged children had the lowest OM hospitalization but the highest TTI rates. This is consistent with findings from a data linkage study conducted elsewhere in Australia [32], that reported similar TTI rates to ours and suggest that socioeconomic status and remoteness rather than Aboriginality explain the disparity [32]. Furthermore, 60% of TTIs in our study were performed in private metropolitan hospitals and, when combined with all metropolitan hospitals, 89% of all TTIs were done privately (S2 Table). These findings highlight the disproportionate provision of services to more advantaged families when the greatest burden is among socio-economically deprived families.

The greatest strength of this study was the ability to investigate OM hospitalizations in a large, unselected population cohort which allowed accurate measurement of rates, increased statistical power and reduced selection bias. In Australia, TTIs are only performed in hospitals on inpatients which allowed us to ascertain all such procedures within this cohort.

When interpreting our data it is important to acknowledge that most OM is diagnosed and treated at the primary care level and not in hospitals. However, population-based administrative datasets at the primary care level are not available. OM-related hospitalizations reported here represent the ‘tip of the iceberg’ and the severe and chronic end of the clinical spectrum.

We had postcode of residence at time of birth but not at time of presentation to hospital. It is unlikely that large proportions of the population move between the broad rural, remote, and metropolitan regions but we cannot discount the fact that some will have migrated between regions. Likewise, it is possible that people residing in disadvantaged postal codes at birth could have moved out to more advantaged areas before hospitalization which may have affected the rates by socio-economic level. By restricting the analyses to younger children we believe we have minimised this limitation.

Future research should focus on assessing the barriers to the detection, referral and surgical management of OM in pre-school Aboriginal children.

## Conclusions

All Aboriginal children and those non-Aboriginal children from lower socio-economic backgrounds were over-represented with OM-related hospitalizations but had fewer TTIs. Future work to reduce the burden of OM among these children should focus on the social determinants of health, particularly the reduction of poverty and increased availability of services especially in rural and remote areas. Data linkage is a robust way to measure rates, and this gives us reason to better understand the differences in hospitalizations between groups of children.

## Supporting information

S1 Fig Rates of hospitalization for principal diagnosis of non-procedural otitis media per 1,000 child-years for non-Aboriginal and Aboriginal children born in Western Australia between 1996–2012. (EPS)

S1 Table List of principal diagnoses (ICD-AM code and description) and number of events when ICD-AM related to otitis media were recorded as additional diagnoses. (DOCX)

S2 Table Tympanostomy tube insertion by hospital type and region of birth for non-Aboriginal and Aboriginal children aged <15 years born between 1996 and 2012 in Western Australia. (DOCX)
