# Supplementary material for: Australian Aboriginal children have higher hospitalization rates for otitis media but lower surgical procedures than non-Aboriginal children: A record linkage population-based cohort study
Source: PLoS One. 2019 Apr 23;14(4):e0215483. doi: 10.1371/journal.pone.0215483 (PMC6478284; doi:10.1371/journal.pone.0215483)
Supplement: S1 Table — (DOCX) [file pone.0215483.s002.docx]

**S1 Table**. List of principal diagnoses (ICD-AM code and description) and number of events when ICD-AM related to otitis media were recorded as additional diagnoses. Only those principal diagnoses with more than 50 observations are detailed below with those <50 in ‘other’ category.

| ICD code | Description | Number of events (%) |
| --- | --- | --- |
| Non-Aboriginal (n=4,821) | | |
| R50.9 | Fever, unspecified | 65 (1) |
| J45.9 | Asthma | 398 (8) |
| J35.0 | Chronic tonsillitis | 86 (2) |
| G47 | Obstructive sleep apnoea | 50 (1) |
| J22 | Acute lower respiratory infection | 150 (3) |
| J21.0, J21.9 | Acute bronchiolitis | 419 (9) |
| J18.0, J18.9 | Pneumonia | 198 (4) |
| J06.9 | Upper respiratory infection | 604 (13) |
| J05.0 | Acute obstructive laryngitis | 79 (2) |
| J03.9 | Acute tonsillitis | 121 (3) |
| B34.9 | Viral infection | 243 (5) |
| A09.0 | Infectious gastroenteritis | 151 (3) |
| A08.4 | Viral intestinal infection | 54 (1) |
| 780.31 | Febrile convulsion | 143 (3) |
|  | Other | 2,060 (43) |
| Aboriginal (n=3,918) | | |
| R62.8 | Delayed milestone | 117 (3) |
| J45.9 | Asthma | 398 (10) |
| J35.0 | Chronic tonsillitis and adenoids | 86 (2) |
| J22 | Acute lower respiratory infection | 201 (5) |
| J21.9 | Acute bronchiolitis | 399 (10) |
| J18.9 | Pneumonia | 246 (6) |
| J06.9 | Upper respiratory tract infection | 407 (10) |
| B34.9 | Viral infection, unspecified | 69 (2) |
| A09.0 | Infectious gastroenteritis | 426 (11) |
| 466.1 | Acute bronchiolitis | 97 (2) |
|  | Other | 1,472 (38) |
