# Supplementary material for: Australian Aboriginal children have higher hospitalization rates for otitis media but lower surgical procedures than non-Aboriginal children: A record linkage population-based cohort study
Source: PLoS One. 2019 Apr 23;14(4):e0215483. doi: 10.1371/journal.pone.0215483 (PMC6478284; doi:10.1371/journal.pone.0215483)
Supplement: S2 Table — (DOCX) [file pone.0215483.s003.docx]

**S2 Table**. Tympanostomy tube insertion by hospital type and region of birth for non-Aboriginal and Aboriginal children aged <15 years born between 1996 and 2012 in Western Australia.

| Non-Aboriginal | | | | Aboriginal | | | |
| --- | --- | --- | --- | --- | --- | --- | --- |
|  | Region of birth | | |  | Region of birth | | |
|  | **Metro n=33,796**  **n(%)** | **Rural**  **n=5,639**  **n(%)** | **Remote**  **n=1,896**  **n(%)** |  | **Metro n=913**  **n(%)** | **Rural**  **n=574**  **n(%)** | **Remote**  **n=766**  **n(%)** |
| **Tertiary or Public Metro** n=11,426 | 10,297 (30.5) | 812 (14.4) | 317 (16.7) | **Tertiary or Public Metro** n=1,094 | 717 (78.5) | 235 (40.9) | 142 (18.5) |
| **Rural** n=3,814 | 473 (1.4) | 2,846 (50.5) | 495 (26.1) | **Rural** n=1,001 | 73 (8.0) | 318 (55.4) | 610 (79.6) |
| **Private Metro** n=26,094 | 23,026 (68.1) | 1,984 (35.2) | 1,084 (57.2) | **Private Metro** n=158 | 123 (13.5) | 21 (3.7) | 14 (1.8) |

53 non-Aboriginal and 19 Aboriginal children not included because of missing residence at birth data.
